# Completed suicide in bipolar i patients after their first hospitalisation

**DOI:** 10.1192/j.eurpsy.2021.1656

**Published:** 2021-08-13

**Authors:** E. Nieto, A. Palau, P. Alvarez, C. Russo

**Affiliations:** Psychiatry, Althaia XarXa Assistencial of Manresa, MANRESA, Spain

**Keywords:** bipolar, Suicide, Hospitalization

## Abstract

**Introduction:**

Bipolar disorder is a mental disorder that has one of the greatest risk of completed suicide (CS)

**Objectives:**

Determine the rate and the risk factors of CS in a cohort of Bipolar I patients followed after their first hospitalization

**Methods:**

We choose all Bipolar I patients (DSM-IV) who were first time hospitalized in our Psychiatric unit between 1996 and 2016. We reviewed the charts of first hospitalization and recorded multiple baseline variables. In the follow-up we updated the database recording all patients who CS. We compared the different baseline variables between Bipolar patients who CS and the rest.

**Results:**

Of a total of 254 bipolar I patients 9 (3,5%) CS in the mean of 13 years of follow up (rate 40 times higher than General Population). The average age at CS was 41.1 years (range between 26 and 71 years old) so there was a 9 years gap on average between the first psychiatric hospitalization and suicide. CS was characterized by a violent act (8 out of 9 cases, 89 %). When we compared BP patients who CS with the rest, only history of suicide in first-degree relatives was detected as a risk factor significantly associated (P<0.01) with CS. Conversely baseline treatment with anticonvulsants (mainly valproate) was detected as a significantly (P<0.004) protective factor of CS.

**Conclusions:**

1-Bipolar I patients after first hospitalization completed suicide 40 times higher than general population almost always by violent method 2-History of CS in first-degree relatives is predictor of completed suicide

**Disclosure:**

No significant relationships.

